# Mortality of Philadelphia, for April, May and June, 1854, Collated from the Record Kept at the Health Office

**Published:** 1854-08

**Authors:** Wilson Jewell


*Mortality of Philadelphia, for April, May and June,* 1854, *collated from the record kept at the Health Office.* By WILSON JEWELL, M. D.

The tabular statement of deaths for the quarter of the year ending the 1st of July, ult., presents the total of 2469; averaging 26 8-10ths deaths daily, or to the population as 1 to every 168.12. An increase of 10 per cent. over the deaths for the same period in 1853.

By deducting the Still Born and the deaths from External Causes, Old Age, Debility and Unknown, amounting in all to 440, it will leave 2029 deaths from accredited diseases.

Exclusive of Still Born, the deaths under one year amount to 574, nearly one fourth the whole number. The deaths recorded under five years from all causes, amount to 1120, or 45 per cent. Those under twenty, to 1365, or 55 per cent.

The excess of deaths among males, amounts to 12 per cent. This excess is observable in all statistics, varying, however, in per centage. Several reasons have been assigned for it, but the true cause is not yet understood. The excess of male still born children is equal to 30 per cent. during the quarter. This excess is also involved in obscurity.

CLASS 1st.—The Endemic or Zymotic diseases exhibit an increase of nine per cent. over those of last year, for the same months. This increase is made up by deaths from Cholera and Measles alone. The comparison shows, that Whooping Cough, Croup, Measles and Diarrhœa, have been on the increase; Scarlet Fever, Erysipelas, Typhus Fever, Dysentery have declined. The total of deaths has been 506. One fourth the whole number were under one year of age.

CLASS 2d.—The diseases of Uncertain seat, number 263. Here there is a slight falling off from those of 1853, same quarter. The uncertainty as to the seat and character of the diseases specified in this class, renders it almost valueless for statistical purposes.

Class 3rd.—"Nervous System." The diseases enumerated under this head, have been augmented twenty-one per cent. over those for the same months of last year. The cases of Inflammation of the Brain have more than doubled themselves. With scarcely an exception, the deaths from all the diseases recorded here have increased; 337, or more than two-thirds, were among children under five years of age. The deaths from Convulsions were 161; of these 144 were children under five years of age.

Class 4th.—"Organs of Respiration." The most prominent diseases in this class, causing death, are Consumption and Inflammation of the Lungs. Of the former, there were 320, more than half of all the deaths caused by diseases of the Respiratory Organs, equal to fifty per cent., or thirteen per cent. of all the deaths that have occurred during the quarter, averaging nearly three and a half deaths per day; the excess is among females, about two per cent. The greatest mortality happened between the ages of 20 and 30 years.

Of Inflammation of the Lungs there were 142, an increase of thirty-nine over those for the same months last year.

The deaths have increased 98 over those of the same class and months for 1853.

Class 5th. "Organs of Circulation." Here the statistics differ but little in numerical distinction from those of the same months in the preceding year. In 1853, the 2d quarter, there were 64. In this year 65. Under one general name, of "Disease of Heart," we find recorded more than one half of all the deaths. A want of correct diagnosis and pathology of disease, on the part of practitioners, characterizes the deaths from this distinctive class, as well as others, elsewhere recorded in these tables.

Class 6th. "Digestive Organs." In this there is a falling off of nearly 2 per cent. from those of last year. Only 127 are noticed, and more than one fourth of these are ascribed to Inflammation of the Stomach and Bowels. In the diseases enumerated under this head, however, there is little if any variation observed from quarter to quarter, except that occasioned by a gradual increase of population.

Class 12th. "External Causes." This embraces accidental and violent deaths. The increase over the same months

1853, is equal to 25 per cent. This augmentation may be accounted for, by the increase in the deaths from Drowning, Intemperance and Asphyxia. The large proportion of deaths among the male population has been alluded to in another place. Eighty-two of the deaths were in the male and only twenty-six in the female sex.

Old Age, under class 11th, furnishes 44 deaths; Still Born 127, and Unknown 66. Under the last head, we infer those cases to be reported, which go through the hands of the coroner, as sudden deaths, &c. It is to be regretted, however, that members in our profession will occasionally be found filling up their certificates with this *omnibus* word, Unknown. This should not occur.

Table 3d, embraces the number of deaths at fifteen distinct periods of human life. This shows the large proportion of deaths among children. Sixty per cent. of the whole number being under 20 years; fifty per cent. under five years, and nearly 30 per cent. under one year. These calculations include the Still Born. The greatest outlet to adult life, appears to be the ages between 20 and 30. Twenty-five per cent. of all the deaths recorded over 20 years, occurring within that decennial period. Three centenarians have died during the quarter.

Since the consolidation of the territory of the whole county under one municipal government, dating from June 19th, the mortuary tables must necessarily be more imperfect than ever. Numerous cemeteries and church burial grounds, scattered through the rural districts, have not been required to make returns to the Board of Health, although the law for several years has embraced the entire county. A nearer approximation to a truthful register of deaths will hereafter, we trust be had, inasmuch as the Board, in its new relation to the city government, are about to require the sextons or trustees of all burial grounds within the county to make weekly returns of their interments. This arrangement, while it will swell the report of deaths, may not increase the per centage to any great extent in proportion to the population, because the population of the entire county will now be the data for the calculation.

TABLE NO. I.

*Deaths for the second quarter of 1854 classified.*

| | Apr. | May | June | Male. A. | Male. M. | Male. J. | Female. A. | Female. M. | Female. J. | Total. |
|---|---|---|---|---|---|---|---|---|---|---|
| 1 *Endemic & Contagious diseases* | | | | | | | | | | |
| Zymotic or Epidemic | 128 | 150 | 228 | 57 | 75 | 111 | 71 | 75 | 117 | 506 |
| 2 *Uncertain or general seat* | | | | | | | | | | |
| Sporadic diseases | 76 | 100 | 87 | 36 | 53 | 43 | 40 | 47 | 44 | 263 |
| 3 Nervous system | 172 | 158 | 172 | 104 | 93 | 103 | 68 | 65 | 69 | 502 |
| 4 Organs of Respiration | 228 | 220 | 165 | 112 | 104 | 86 | 116 | 116 | 79 | 613 |
| 5 " Circulation | 22 | 21 | 22 | 12 | 11 | 11 | 10 | 10 | 11 | 65 |
| 6 Digestive organs | 37 | 53 | 37 | 22 | 26 | 24 | 15 | 27 | 13 | 127 |
| 7 Urinary " | 4 | 1 | 5 | 5 | 1 | 3 | | | 1 | 10 |
| 8 Organs of Generation | 3 | 9 | 8 | | | | 3 | 9 | 8 | 20 |
| 9 " Locomotion | 4 | 3 | 5 | 3 | 2 | 3 | 1 | 1 | 2 | 12 |
| 10 Integumentary system | 2 | 4 | | 1 | 4 | | 1 | | | 6 |
| 11 Old age | 15 | 16 | 13 | 5 | 5 | 6 | 10 | 11 | 7 | 44 |
| 12 External causes | 25 | 48 | 35 | 17 | 37 | 28 | 8 | 11 | 7 | 108 |
| Still Born | 42 | 43 | 42 | 26 | 25 | 24 | 16 | 18 | 18 | 127 |
| Unknown | 13 | 34 | 19 | 7 | 21 | 11 | 6 | 13 | 8 | 66 |
| | 771 | 860 | 838 | 407 | 457 | 453 | 365 | 403 | 384 | 2469 |

TABLE NO. 2.

1. *Endemic and Contagious Diseases—Zymotic or Epidemic.*

| | Male. | Female. | Under 1 yr. | 1 to 2. | 2 to 5. | 5 to 10. | 10 to 15. | 15 to 20. | 20 to 30. | 30 to 40. | 40 to 50. | 50 to 60. | 60 to 70. | 70 to 80. | 80 to 90. | 90 to 100. | 100 to 110. | Total. |
|---|---|---|---|---|---|---|---|---|---|---|---|---|---|---|---|---|---|---|
| Cholera | 18 | 5 | | | | | 1 | 1 | 9 | 8 | 2 | 2 | | | | | | 23 |
| " infantum | 20 | 43 | 47 | 10 | 6 | | | | | | | | | | | | | 63 |
| " morbus | 5 | 5 | 4 | | 1 | | | | 1 | 1 | 1 | 1 | 1 | | | | | 10 |
| Croup | 35 | 47 | 15 | 21 | 35 | 11 | | | | | | | | | | | | 82 |
| Diarrhœa | 20 | 11 | 10 | 2 | 2 | 1 | 2 | 1 | 4 | 2 | 4 | 1 | 1 | 1 | | | | 31 |
| Dysentery | 24 | 22 | 7 | 11 | 11 | 2 | | 2 | 2 | | 2 | 3 | 5 | 1 | | | | 46 |
| Erysipelas | 13 | 8 | 5 | 2 | 1 | | | | 4 | 3 | 1 | 3 | 1 | 1 | | | | 21 |
| Fever, | 4 | 2 | | 1 | | 1 | | | 2 | | 1 | 1 | | | | | | 6 |
| " Bilious | | 1 | | | | | | | | | | | | 1 | | | | 1 |
| " " Remittent | 1 | | | | | | | | 1 | | | | | | | | | 1 |
| " Congestive | 1 | 1 | | | | | | 2 | | | | | | | | | | 2 |
| " Continued | | 1 | | | | | | | | | | 1 | | | | | | 1 |
| " Intermittent | | 1 | | | | | | | | | | | | 1 | | | | 1 |
| " Nervous | 1 | | | | | | | | | | | | 1 | | | | | 1 |
| " Puerperal | | 8 | | | | | | | 5 | 2 | 1 | | | | | | | 8 |
| " Remittent | 5 | 5 | | 3 | 3 | 4 | | | | | | | | | | | | 10 |
| " Scarlet | 17 | 23 | 2 | 7 | 23 | 7 | 1 | | | | | | | | | | | 43 |
| " Typhoid | 22 | 22 | | 3 | 3 | 4 | 2 | 5 | 7 | 12 | 1 | 6 | | 1 | | | | 44 |
| " Typhus | 15 | 5 | | | | 2 | | 1 | 8 | 1 | 4 | | 1 | 3 | | | | 20 |
| Hooping Cough | 17 | 28 | 24 | 14 | 5 | 2 | | | | | | | | | | | | 45 |
| Influenza | 1 | 2 | | 1 | | | | | | | | | | 1 | 1 | | | 3 |
| Measles | 14 | 17 | 8 | 11 | 9 | 1 | | 1 | | 1 | | | | | | | | 31 |
| Small Pox | 10 | 6 | 5 | 3 | | 3 | 1 | 1 | 3 | | | | | | | | | 16 |
| | 243 | 263 | 127 | 89 | 99 | 38 | 7 | 14 | 46 | 30 | 17 | 18 | 10 | 10 | 1 | | | 506 |

2. *Uncertain or General Seat—Sporadic Diseases.*

| | Male. | Female. | Under 1 yr. | 1 to 2. | 2 to 5. | 5 to 10. | 10 to 15. | 15 to 20. | 20 to 30. | 30 to 40. | 40 to 50. | 50 to 60. | 60 to 70. | 70 to 80. | 80 to 90. | 90 to 100. | 100 to 110. | Total. |
|---|---|---|---|---|---|---|---|---|---|---|---|---|---|---|---|---|---|---|
| Abscess Face | 1 | | | 1 | | | | | | | | | | | | | | 1 |
| " Lumbar | 1 | | | | | | | | | | | 1 | | | | | | 1 |
| " Parotid Gland | | 1 | | | | | | | 1 | | | | | | | | | 1 |
| " Psoas | | 1 | | | | 1 | | | | | | | | | | | | 1 |
| Cancer | 1 | 4 | | 1 | | | | | | 1 | 1 | 2 | | | | | | 5 |
| " Breast | | 1 | | | | | | | | | | | | | 1 | | | 1 |
| Cachexia | | 1 | 1 | | | | | | | | | | | | | | | 1 |
| Caries | 1 | | | | | | | 1 | | | | | | | | | | 1 |
| Debility | 49 | 46 | 51 | 2 | 2 | 1 | | | 2 | 7 | 1 | 8 | 10 | 5 | 5 | 1 | | 95 |
| Dropsy | 10 | 10 | | 2 | 2 | 2 | | 1 | 2 | 1 | 1 | 2 | 3 | 2 | 2 | | | 20 |
| Effusion | | 1 | | | 1 | | | | | | | | | | | | | 1 |
| Enlargem't cervical glands | | 1 | | | | 1 | | | | | | | | | | | | 1 |
| Gangrene | 2 | 1 | | | 1 | | | | | | | | 1 | 1 | | | | 3 |
| " Mouth | | 1 | | | 1 | | | | | | | | | | | | | 1 |
| Hemorrhage | 6 | 2 | 3 | | | | | 2 | 2 | 1 | | | | | | | | 8 |
| Inanition | 8 | 5 | 10 | 1 | | | | | | 1 | | | | 1 | | | | 13 |
| Inflammation | 1 | | | 1 | | | | | | | | | | | | | | 1 |
| " Parotid Gland | | 1 | | | | | | | 1 | | | | | | | | | 1 |
| " Throat | 2 | | | | 1 | | | | 1 | | | | | | | | | 2 |
| Malformation | 4 | 3 | 7 | | | | | | | | | | | | | | | 7 |
| Marasmus | 40 | 45 | 58 | 6 | 10 | 3 | | | 2 | 1 | 4 | | | 1 | | | | 85 |
| Mortification | 1 | | | | | | | | | 1 | | | | | | | | 1 |
| Scrofula | 3 | 4 | 2 | | 1 | 1 | 1 | | 1 | | | 1 | | | | | | 7 |
| Tabes Mesenterica | | 2 | | | 1 | 1 | | | | | | | | | | | | 2 |
| Spina Bifida | 1 | | 1 | | | | | | | | | | | | | | | 1 |
| Tumour | 1 | 1 | | | 1 | | | | 1 | | | | | | | | | 2 |
| | 132 | 131 | 133 | 14 | 21 | 10 | 1 | 4 | 13 | 13 | 7 | 14 | 14 | 10 | 8 | 1 | | 263 |

3. *Nervous Diseases.*

| | Male. | Female. | Under 1 yr. | 1 to 2. | 2 to 5. | 5 to 10. | 10 to 15. | 15 to 20. | 20 to 30. | 30 to 40. | 40 to 50. | 50 to 60. | 60 to 70. | 70 to 80. | 80 to 90. | 90 to 100. | 100 to 110. | Total. |
|---|---|---|---|---|---|---|---|---|---|---|---|---|---|---|---|---|---|---|
| Abscess of Brain | 2 | 1 | | 2 | | | | | | 1 | | | | | | | | 3 |
| Apoplexy | 14 | 4 | | | | | 1 | | 1 | 3 | 1 | 5 | 5 | 1 | 1 | | | 18 |
| Chorea | | 2 | | | | | 1 | | | 1 | | | | | | | | 2 |
| Concussion of Brain | 2 | 1 | | | | | | | 1 | 1 | | 1 | | | | | | 3 |
| Congestion " | 25 | 15 | 7 | 5 | 6 | 5 | 1 | 2 | 4 | 4 | 2 | | 2 | 1 | 1 | | | 40 |
| Convulsions | 92 | 69 | 100 | 31 | 13 | 3 | | | 2 | 2 | 4 | 1 | 3 | 2 | | | | 161 |
| Disease of Brain | 9 | 12 | 4 | 2 | 7 | 3 | 2 | | 2 | | | | | 1 | | | | 21 |
| Dropsy " | 49 | 32 | 30 | 25 | 20 | 4 | | 1 | 1 | | | | | | | | | 81 |
| Effusion " | 12 | 8 | 5 | 7 | 3 | 1 | | | | 1 | 2 | | 1 | | | | | 20 |
| Epilepsy | 4 | | 1 | | | | | | | | 1 | 2 | | | | | | 4 |
| Hemorrhage from Brain | | 1 | | | | | | | | | | | | 1 | | | | 1 |
| Hypochondritis | 1 | | | | | | | | | 1 | | | | | | | | 1 |
| Inflammation of Brain | 62 | 35 | 24 | 14 | 23 | 15 | 4 | 2 | 5 | 4 | 4 | 1 | | 1 | | | | 97 |
| Irritation of Brain | | 1 | | | | | | | | | | | | 1 | | | | 1 |
| Mania | 2 | 1 | | | | | | | 1 | 1 | 1 | | | | | | | 3 |
| " a Potu | 9 | 2 | | | | | | | | 4 | 4 | 3 | | | | | | 11 |
| " Puerperal | | 1 | | | | | | | | 1 | | | | | | | | 1 |
| Palsy | 8 | 15 | 1 | | | 1 | | 1 | 2 | | 3 | 1 | 4 | 5 | 3 | 2 | | 23 |
| Softening of Brain | 3 | | | | | | | | | | 1 | | 2 | | | | | 3 |
| Tetanus | 3 | | 1 | | | | | | 1 | | | | 1 | | | | | 3 |
| Thymic Asthma | 2 | 1 | 3 | | | | | | | | | | | | | | | 3 |
| Trismus | 1 | 1 | 2 | | | | | | | | | | | | | | | 2 |
| | 300 | 202 | 178 | 86 | 73 | 32 | 9 | 6 | 20 | 24 | 23 | 14 | 18 | 12 | 5 | 2 | | 502 |

*4. Organs of Respiration.*

| | Male. | Female. | Under 1 yr. | 1 to 2. | 2 to 5. | 5 to 10. | 10 to 15. | 15 to 20. | 20 to 30. | 30 to 40. | 40 to 50. | 50 to 60. | 60 to 70. | 70 to 80. | 80 to 90. | 90 to 100. | 100 to 110. | Total. |
|---|---|---|---|---|---|---|---|---|---|---|---|---|---|---|---|---|---|---|
| Abscess of Lungs | 2 | | | | | | 1 | | | | 1 | | | | | | | 2 |
| Asthma | 4 | 3 | 3 | | | | | | | | 1 | 2 | | | 1 | | | 7 |
| Congestion of Lungs | 6 | 8 | 1 | 2 | 5 | | | | 2 | 2 | | 1 | 1 | | | | | 14 |
| Consumption " | 144 | 176 | 6 | 2 | 14 | 9 | 7 | 26 | 97 | 61 | 44 | 32 | 14 | 6 | 1 | | 1 | 320 |
| Coryza | 1 | | 1 | | | | | | | | | | | | | | | 1 |
| Disease of Chest | 2 | 2 | | | 1 | 1 | 1 | | | | | 1 | | | | | | 4 |
| " Lungs | 4 | 4 | | | 1 | 1 | | 1 | 1 | 1 | | 1 | 1 | 1 | | | | 8 |
| Dropsy of Chest | 8 | 2 | | | | | | | 1 | 2 | 1 | 1 | 2 | 2 | | | 1 | 10 |
| Effusion of Lungs | 1 | 2 | | 1 | | 1 | | | | | | 1 | | | | | | 3 |
| " Chest | 1 | | | | 1 | | | | | | | | | | | | | 1 |
| Empyema | | 1 | | | | | | | | | | 1 | | | | | | 1 |
| Fever Hectic | 1 | 2 | | | 2 | | | | | | 1 | | | | | | | 3 |
| Gangrene of Lungs | 1 | | | | | | | | | | | 1 | | | | | | 1 |
| Hemorrhage of " | 5 | 2 | | | | | | 1 | 4 | | 1 | | 1 | | | | | 7 |
| Inflamm'n of Bronchiæ | 31 | 33 | 24 | 20 | 7 | 3 | | | 4 | 2 | 1 | | 1 | | 2 | | | 64 |
| " Breast | | 1 | | | 1 | | | | | | | | | | | | | 1 |
| " Chest | 1 | 2 | 2 | | | | | 1 | | | | | | | | | | 3 |
| " Larynx | 1 | | | | | | | | | | | 1 | | | | | | 1 |
| " Lungs | 80 | 62 | 27 | 34 | 26 | 10 | | 2 | 5 | 9 | 9 | 7 | 8 | 3 | 2 | | | 142 |
| " Pleura | 3 | 4 | 1 | | 1 | | | | | 1 | 1 | 1 | 2 | | | | | 7 |
| Tuberculosis | 5 | 5 | 2 | | | | | 2 | | 4 | 1 | 1 | | | | | | 10 |
| Ulceration of Larynx | 1 | 1 | | | 1 | | | | | 1 | | | | | | | | 2 |
| " Lungs | | 1 | | | 1 | | | | | | | | | | | | | 1 |
| | 302 | 311 | 67 | 59 | 61 | 25 | 9 | 33 | 114 | 83 | 61 | 51 | 30 | 12 | 6 | | 2 | 613 |

*5. Organs of Circulation.*

| | Male. | Female. | Under 1 yr. | 1 to 2. | 2 to 5. | 5 to 10. | 10 to 15. | 15 to 20. | 20 to 30. | 30 to 40. | 40 to 50. | 50 to 60. | 60 to 70. | 70 to 80. | 80 to 90. | 90 to 100. | 100 to 110. | Total. |
|---|---|---|---|---|---|---|---|---|---|---|---|---|---|---|---|---|---|---|
| Anæmia | | 3 | | | | | | | 1 | | 1 | | 1 | | | | | 3 |
| Aneurism of Aorta | 1 | | | | | | | | | | 1 | | | | | | | 1 |
| Cyanosis | 4 | 2 | 6 | | | | | | | | | | | | | | | 6 |
| Disease of Heart | 20 | 17 | 3 | | 2 | 3 | 1 | 3 | 6 | 7 | 2 | 4 | 3 | 2 | 1 | | | 37 |
| Dropsy " | | 2 | | | | | | | 1 | | 1 | | | | | | | 2 |
| Effusion " | 1 | | | | 1 | | | | | | | | | | | | | 1 |
| Enlargem't " | 2 | 3 | | | | | | 1 | 1 | | 2 | 1 | | | | | | 5 |
| Inflammat'n " | 2 | 1 | | | | 1 | 1 | | 1 | | | | | | | | | 3 |
| Malformation of Heart | 2 | 3 | 4 | 1 | | | | | | | | | | | | | | 5 |
| Rheumatism " | 1 | | | | | | | 1 | | | | | | | | | | 1 |
| Softening " | 1 | | | | | | | | | | | | 1 | | | | | 1 |
| | 34 | 31 | 13 | 1 | 3 | 4 | 2 | 5 | 10 | 7 | 7 | 5 | 5 | 2 | 1 | | | 65 |

6. *Digestive Organs.*

| | Male. | Female. | Under 1 yr. | 1 to 2. | 2 to 5. | 5 to 10. | 10 to 15. | 15 to 20. | 20 to 30. | 30 to 40. | 40 to 50. | 50 to 60. | 60 to 70. | 70 to 80. | 80 to 90. | 90 to 100. | 100 to 110. | Total. |
|---|---|---|---|---|---|---|---|---|---|---|---|---|---|---|---|---|---|---|
| Abscess of Liver | 2 | 1 | 1 | | | | | | | 2 | | | | | | | | 3 |
| Cancer of Stomach | 2 | 1 | | | | | | | 1 | 1 | | 1 | | | | | | 3 |
| Cancrum Oris | 2 | 3 | | | 3 | 1 | | | 1 | | | | | | | | | 5 |
| Cramp of Stomach | 1 | | 1 | | | | | | | | | | | | | | | 1 |
| Cirrhosis of Liver | 4 | | | | | 1 | | | | | 1 | 1 | 1 | | | | | 4 |
| Congestion of Bowels | 1 | 1 | 1 | 1 | | | | | | | | | | | | | | 2 |
| " Liver | 1 | | | | | | | | | | | 1 | | | | | | 1 |
| Consumption of Liver | 2 | | 1 | | | 1 | | | | | | | | | | | | 2 |
| Disease of Bowels | 1 | 1 | 1 | | | | | | | 1 | | | | | | | | 2 |
| " Liver | | 1 | | | | | | | | | | | | 1 | | | | 1 |
| " Stomach | | 1 | | | | | | | | | 1 | | | | | | | 1 |
| Dropsy, Abdominal | 8 | 4 | 1 | | | | 2 | 2 | | 2 | | 1 | 3 | 1 | | | | 12 |
| Dyspepsia | 2 | | | | | | | 1 | | | 1 | | | | | | | 2 |
| Enlargement of Liver | | 1 | | | | | | | | | | | 1 | | | | | 1 |
| Hemorrhage of Bowels | 1 | | | | | | | | | | | 1 | | | | | | 1 |
| Hernia, Strangulated | 1 | 1 | | | | | | | 1 | | | | 1 | | | | | 2 |
| " Umbilical | | 1 | 1 | | | | | | | | | | | | | | | 1 |
| Hernia | | 1 | | | | | | | | | | | | | 1 | | | 1 |
| Inflammation of Intestines | 1 | | | | | | | | 1 | | | | | | | | | 1 |
| " Liver | 2 | 8 | | | | 1 | | | | 3 | 3 | 1 | 2 | | | | | 10 |
| " Peritoneum | 5 | 6 | | | | | | 2 | 3 | 5 | 1 | | | | | | | 11 |
| " Spleen | | 1 | | | | | | | | 1 | | | | | | | | 1 |
| " Stom. & Bowels | 23 | 12 | 9 | 8 | 5 | 1 | | 1 | 2 | | 4 | 3 | 2 | | | | | 35 |
| " Tonsils | | 1 | | | | | | | | | | 1 | | | | | | 1 |
| Ileus | | 1 | | | | | | | | | 1 | | | | | | | 1 |
| Intussusception | 1 | 1 | | | | | 1 | 1 | | | | | | | | | | 2 |
| Jaundice | 3 | 1 | 2 | | | 1 | | | | | 1 | | | | | | | 4 |
| Melanosis | | 1 | | | | | | | 1 | | | | | | | | | 1 |
| Mortification of Bowels | | 1 | | | | | | | | | 1 | | | | | | | 1 |
| Obstruction of Bowels | 1 | | | | | | | | | | | 1 | | | | | | 1 |
| Schirrus Stomach | 1 | | | | | | | | | | | 1 | | | | | | 1 |
| Teething | 3 | 1 | 2 | 1 | 1 | | | | | | | | | | | | | 4 |
| Tympanitis | | 2 | 1 | | | | | | | | | | 1 | | | | | 2 |
| Ulceration of Bowels | 3 | 2 | | 1 | 1 | | | | | 1 | 1 | | | | 1 | | | 5 |
| " Colon | 1 | | | | | | | | 1 | | | | | | | | | 1 |
| | 72 | 55 | 21 | 11 | 10 | 6 | 3 | 7 | 11 | 16 | 15 | 12 | 11 | 2 | 2 | | | 127 |

7. *Urinary Organs.*

| | Male. | Female. | Under 1 yr. | 1 to 2. | 2 to 5. | 5 to 10. | 10 to 15. | 15 to 20. | 20 to 30. | 30 to 40. | 40 to 50. | 50 to 60. | 60 to 70. | 70 to 80. | 80 to 90. | 90 to 100. | 100 to 110. | Total. |
|---|---|---|---|---|---|---|---|---|---|---|---|---|---|---|---|---|---|---|
| Albuminuria | 1 | | | | | | | | | 1 | | | | | | | | 1 |
| Cancer of Bladder | 1 | | | | | | | | | | | | 1 | | | | | 1 |
| Diabetes | 1 | | | | | | | | | | | 1 | | | | | | 1 |
| Disease of Kidneys, | 4 | 1 | | | | | | | | 2 | | 1 | 1 | 1 | | | | 5 |
| Inflammation of Bladder, | 2 | | | | | | | | 1 | | 1 | | | | | | | 2 |
| | 9 | 1 | | | | | | | 1 | 3 | 1 | 2 | 2 | 1 | | | | 10 |

8. *Organs of Generation.*

| | Male. | Female. | Under 1 yr. | 1 to 2. | 2 to 5. | 5 to 10. | 10 to 15. | 15 to 20. | 20 to 30. | 30 to 40. | 40 to 50. | 50 to 60. | 60 to 70. | 70 to 80. | 80 to 90. | 90 to 100. | 100 to 110. | Total. |
|---|---|---|---|---|---|---|---|---|---|---|---|---|---|---|---|---|---|---|
| Cancer of Uterus | | 4 | | | | | | | | 2 | 1 | | | 1 | | | | 4 |
| Child Bed, | | 3 | | | | | | | 2 | 1 | | | | | | | | 3 |
| Convulsions, Puerperal | | 3 | | | | | | | 1 | 2 | | | | | | | | 3 |
| Disease of Uterus | | 3 | | | | | | | 1 | 1 | | | 1 | | | | | 3 |
| Dropsy of Ovaries | | 1 | | | | | | | | | | | | 1 | | | | 1 |
| Hemorrhage of Uterus | | 2 | | | | | | | | 1 | 1 | | | | | | | 2 |
| Inflammation of " | | 2 | | | | | | | 1 | | 1 | | | | | | | 2 |
| " of Ovaries | | 1 | | | | | | | | | 1 | | | | | | | 1 |
| Tumour of Uterus | | 1 | | | | | | | | | | 1 | | | | | | 1 |
| | | 20 | | | | | | | 5 | 7 | 4 | 1 | 1 | 2 | | | | 20 |

9. *Organs of Locomotion.*

| | Male. | Female. | Under 1 yr. | 1 to 2. | 2 to 5. | 5 to 10. | 10 to 15. | 15 to 20. | 20 to 30. | 30 to 40. | 40 to 50. | 50 to 60. | 60 to 70. | 70 to 80. | 80 to 90. | 90 to 100. | 100 to 110. | Total. |
|---|---|---|---|---|---|---|---|---|---|---|---|---|---|---|---|---|---|---|
| Caries of Spine | 1 | | | | | | | 1 | | | | | | | | | | 1 |
| Compression of Spinal Marrow | 1 | | | | | | | | 1 | | | | | | | | | 1 |
| Curvature of Spine | | 1 | | | | | | | | | 1 | | | | | | | 1 |
| Disease of " | 3 | 2 | 1 | 1 | | 1 | 1 | 1 | | | | | | | | | | 5 |
| Rheumatism | 2 | 1 | | | | | | 1 | 1 | | | | | | | | | 3 |
| Softening of Spine | 1 | | | | | | | | | 1 | | | 1 | | | | | 1 |
| | 8 | 4 | 1 | 1 | | 1 | 1 | 3 | 2 | 1 | 1 | | 1 | | | | | 12 |

10. *Integumentary System.*

| | Male. | Female. | Under 1 yr. | 1 to 2. | 2 to 5. | 5 to 10. | 10 to 15. | 15 to 20. | 20 to 30. | 30 to 40. | 40 to 50. | 50 to 60. | 60 to 70. | 70 to 80. | 80 to 90. | 90 to 100. | 100 to 110. | Total. |
|---|---|---|---|---|---|---|---|---|---|---|---|---|---|---|---|---|---|---|
| Carbuncle | 1 | | | | | | | | | | | 1 | | | | | | 1 |
| Purpura Hemorrhagica | 4 | 1 | 3 | | 1 | | | | | | | | 1 | | | | | 5 |
| | 5 | 1 | 3 | | 1 | | | | | | | 1 | 1 | | | | | 6 |

11. *Old Age.*

| | | | | | | | | | | | | | | | | | | |
|---|---|---|---|---|---|---|---|---|---|---|---|---|---|---|---|---|---|---|
| Old Age | 16 | 28 | | | | | | | | | | | 4 | 20 | 14 | 5 | 1 | 44 |

12. *External Causes.*

| | Male. | Female. | Under 1 yr. | 1 to 2. | 2 to 5. | 5 to 10. | 10 to 15. | 15 to 20. | 20 to 30. | 30 to 40. | 40 to 50. | 50 to 60. | 60 to 70. | 70 to 80. | 80 to 90. | 90 to 100. | 100 to 110. | Total. |
|---|---|---|---|---|---|---|---|---|---|---|---|---|---|---|---|---|---|---|
| Asphyxia | 3 | 6 | 8 | 1 | | | | | | | | | | | | | | 9 |
| Burns | 2 | 8 | 1 | 2 | 6 | | | | | | | 1 | | | | | | 10 |
| Casualties | 28 | 2 | 1 | | 1 | 2 | 1 | 2 | 7 | 10 | 3 | 1 | 2 | | | | | 30 |
| Drowned | 33 | 1 | | | | 2 | 4 | 7 | 6 | 2 | 13 | | | | | | | 34 |
| Fracture of Arm | 1 | | | | | | | | 1 | | | | | | | | | 1 |
| " Leg | 1 | | | | | | | | | | 1 | | | | | | | 1 |
| " Skull | 2 | | | | 1 | | | | | | 1 | | | | | | | 2 |
| " Thigh | 1 | | | | | | | | | | | | 1 | | | | | 1 |
| Hydrophobia | 1 | | | | | | 1 | | | | | | | | | | | 1 |
| Intemperance | 8 | 7 | | | | | | | 1 | 5 | 5 | 2 | 2 | | | | | 15 |
| Poisoning | 1 | 1 | 1 | | | | | | 1 | | | | | | | | | 2 |
| Strangulation | 1 | | | | | | | | | | 1 | | | | | | | 1 |
| Suicide | | 1 | | | | | | | | 1 | | | | | | | | 1 |
| | 82 | 26 | 11 | 3 | 8 | 4 | 6 | 9 | 16 | 18 | 24 | 4 | 5 | | | | | 108 |
| Still Born | 75 | 52 | 127 | | | | | | | | | | | | | | | 127 |
| Unknown | 39 | 27 | 20 | 3 | 3 | 3 | 1 | 2 | 5 | 8 | 7 | 3 | 7 | 3 | 1 | | | 66 |

TABLE NO. 3.

*Deaths for the Second Quarter of 1854, at fifteen distinct periods of life.*

| | |
|---|---|
| Under 1 year, | 574 |
| 1 to 2 | 267 |
| 2 to 5 | 279 |
| 5 to 10 | 123 |
| 10 to 15 | 39 |
| 15 to 20 | 83 |
| 20 to 30 | 243 |
| 30 to 40 | 210 |
| 40 to 50 | 167 |
| 50 to 60 | 125 |
| 60 to 70 | 109 |
| 70 to 80 | 74 |
| 80 to 90 | 38 |
| 90 to 100 | 8 |
| 100 to 110 | 3 |
| | 2342 |
| Still Born | 127 |
| Total, | 2469 |

Embraced within the above table, were 177 from the Blockley Alms House; 165 Blacks, and 10 from the country, as follows:

| | April. | May. | June. | Total. |
|---|---|---|---|---|
| Almshouse, | 45 | 73 | 59 | 177 |
| Blacks, | 54 | 65 | 46 | 165 |
| Country, | 3 | 2 | 5 | 10 |
| Total, | | | | 352 |

